# *Ascophyllum nodosum* Extract (Sealicit^TM^) Boosts Soybean Yield Through Reduction of Pod Shattering-Related Seed Loss and Enhanced Seed Production

**DOI:** 10.3389/fpls.2021.631768

**Published:** 2021-02-24

**Authors:** Łukasz Łangowski, Oscar Goñi, Fabio Serafim Marques, Osvaldo Toshiyuki Hamawaki, Carolina Oliveira da Silva, Ana Paula Oliveira Nogueira, Morgana Aparecida Justino Teixeira, Jacqueline Siqueira Glasenapp, Marcio Pereira, Shane O’Connell

**Affiliations:** ^1^Brandon Bioscience, Tralee, Ireland; ^2^Plant Biostimulant Group, Shannon Applied Biotechnology Centre, Munster Technological University Kerry, Tralee, Ireland; ^3^Instituto de Ciências Agrárias, Universidade Federal de Uberlândia/UFU, Uberlândia, Brazil; ^4^Instituto de Biotecnologia, Universidade Federal de Uberlândia/UFU, Uberlândia, Brazil; ^5^Fundação Educacional de Ituverava FAFRAM, Faculdade Agronomia, Ituverava, Brazil

**Keywords:** seaweed, *Ascophyllum nodosum* extract, pod shattering, soybean (*Glycine max*), seed loss, sustainable agriculture, plant biostimulants

## Abstract

Soybean is one of the most valuable commercial crops because of its high protein, carbohydrate, and oil content. The land area cultivated with soybean in subtropical regions, such as Brazil, is continuously expanding, in some instances at the expense of carbon storing natural habitats. Strategies to decrease yield/seed losses and increase production efficiency are urgently required to meet global demand for soybean in a sustainable manner. Here, we evaluated the effectiveness of an *Ascophyllum nodosum* extract (ANE), Sealicit^TM^, in increasing yields of different soybean varieties, in two geographical regions (Canada and Brazil). In addition, we investigated the potential of Sealicit^TM^ to reduce pod shattering at the trials in Brazil. Three different concentrations of Sealicit^TM^ were applied to pod shatter-susceptible (SS) UFUS 6901 and shatter-resistant (SR) UFUS 7415 varieties to assess their impact on pod firmness. SS variety demonstrated a significant decrease in pod shattering, which coincided with deregulation of *GmPDH1.1* and *GmSHAT1–5* expression, genes that determine pod dehiscence, and higher seed weight per pod. Sealicit^TM^ application to the SR variety did not significantly alter its inherent pod shatter resistance, but provided higher increases in seed yield at harvest. This yield increase maybe associated with to other yield components stimulated by the biostimulant. This work demonstrates that Sealicit^TM^, which has previously been shown to improve pod firmness in *Arabidopsis* and selected commercial oilseed rape varieties through *IND* gene down-regulation, also has the potential to improve pod resistance and seed productivity in soybean, a member of the legume family sharing a similar strategy for seed dispersal.

## Introduction

The increased food production requirement, in the context of limited availability of arable land and a climate emergency driven by greenhouse gas emissions, makes the development of more efficient agricultural production a necessity. At the same time, consumer trends for consumption of more plant protein and less meat result in surging demand for plant proteins and oils that are very efficiently produced by legumes, especially soybean. Since 1961, the world production of soybean has increased 13-fold through the rapid expansion of the production area (from 23.8 to 124.9 Mha) and a significant boost of average harvested seed yield (from 1,128 to 2,791 kg/ha). In 2018, United States (35%), Brazil (34%), and Argentina (11%) produced approximately 80% of the world’s harvested soybean, which is equivalent to almost 350 Mt (FAO, 2018) and steadily growing.

Soybean (*Glycine max*) is a relatively young crop; it is estimated that it was domesticated only 3,000–5,000 years ago in China (Prince et al., 2020), with subsequent cultivation spreading to other Asian regions. It is believed that during that time soybean acquired a number of shatter-resistant genes to impair an evolutionary-conserved seed dispersal mechanism (Dong et al., 2014). Current crops have been optimized for desired traits, starting from the selection by first farmers (Meng et al., 2013), through genome-wide association analysis (GWAS) combined with bioinformatics (Moreira et al., 2019). Independently of the method, the decrease of seed loss by dispersal and maximization of seed production was always the main goal. The major discoveries revealing the mechanism of seed dispersal are mostly attributed to the acquisition of *Arabidopsis thaliana* as a model plant system for fundamental research. *Arabidopsis*, a member of the Brassicaceae, develops a characteristic dry dehiscent fruit composed of two fused carpels that shatter the seeds through breaking of the dehiscence zones, along the silique, after maturity (Ferrándiz, 2002; Łangowski et al., 2016). Apart from *Arabidopsis*, several important genes involved in pod shattering, namely, *IND*, *ALC*, *SHP1*, *SHP2*, and *FUL*, and their complex regulatory network involved in pod dehiscence, were identified in a number of other members of the *brassicas* (Liljegren et al., 2000, 2004; Rajani and Sundaresan, 2001; Lewis et al., 2006; Girin et al., 2011; Pabón-Mora et al., 2014; Balanzà et al., 2016) such as *Capsella rubella* (Eldridge et al., 2016; Dong et al., 2019) and *Brassica juncea* (Østergaard et al., 2006), *rapa* (Mongkolporn et al., 2003), or *napus* (Raman et al., 2014), as well as in rice and soybean (Konishi et al., 2006; Li et al., 2006; Suzuki et al., 2010; Funatsuki et al., 2014; Yoon et al., 2014; Dong and Wang, 2015). In contrast to the bicarpellate silique of crucifers, legumes (including soybean) develop a fruit consisting of a single carpel fused on both sides. The pod walls, composed of several layers and cell types, are connected along the ventral and dorsal sutures. The opening of the soybean pod is triggered by the tension built inside the senescing pod and starts on the dorsal side (Tiwari and Bhatia, 1995; Zhang et al., 2018). Dong et al. (2014) found that excessive lignification of fiber cap cells (FCC) conferred the shatter-resistant phenotype. This process is controlled by SHATTERING1–5 (SHAT1–5), homologs to AtNST1/2, which is known to promote secondary wall biosynthesis. The same study compared gene expression between the wild allele and domesticated soybean, revealing that *SHAT1–5* was 15-fold higher expressed than its wild counterpart. In parallel, Funatsuki et al. (2014) reported another major QTL (quantitative trait locus) involved in soybean pod shattering. Complementation assays showed that *POD DEHISCENCE1* (*GmPDH1.1*) was found to be highly expressed in inner sclerenchyma, which correlated with lignin deposition. *GmPDH1.1* encodes a dirigent-like protein involved in lignin biosynthesis (Suzuki et al., 2010) and pod dehiscence by increasing the twisting force in the pod wall (Dong and Wang, 2015). Therefore, the loss-of-function of *Gmpdh1* has been widely used as a shattering-resistant gene in soybean breeding (Funatsuki et al., 2014). Additionally, more than half of Chinese landraces and most of South Asian landraces were shown to possess *PDH1* and its shatter-resistant *pdh1* allele, which suggested that *pdh1* was indispensable to effectively grow soybean in a dry climate (Kaga et al., 2012; Funatsuki et al., 2014). The soybean pod is designed to withstand the natural fluctuations of humidity and temperature; however, the longer it stays in the field and the more cycles of wetting/drying occur, the higher the chance the pods will burst open with loss of the seeds to the soil. Unfortunately, because of rapidly changing weather conditions, with unexpectedly long periods of drought or rainfall and therefore delayed harvest, soybean shattering in the field, or during harvest, are becoming more frequent. These factors have been directly correlated to higher losses (Menezes et al., 2018; Zhang et al., 2018; Silveira et al., 2019), averaging 120 kg/ha in Brazil (Schanoski et al., 2011), or up to 319 kg/ha in the Southeastern United States (Philbrook and Oplinger, 1989).

In the last decade, a class of crop inputs known as plant biostimulants has gained significant attention for improving plant productivity. Plant biostimulants, especially those extracted from the brown alga *Ascophyllum nodosum*, have been reported to deliver a number of benefits to plants/crops (Craigie, 2011; Vera et al., 2011; Sharma et al., 2013; Ali et al., 2016; Goñi et al., 2016, 2018; Shukla et al., 2018, 2019; Carmody et al., 2020). Previously, we reported the efficacy of a specialty *A. nodosum* extract (ANE) biostimulant, namely, Sealicit^TM^, and explored the mode of action (MOA) in reducing pod dehiscence in *A. thaliana* and winter oilseed rape (WOSR) (Łangowski et al., 2019). Sealicit^TM^ is a novel ANE containing PSI-759 biomolecule complex, produced using a targeted plant signal induction (PSI) approach to formulation development. In this study, the goal was to test the efficacy of Sealicit^TM^ in increasing yield and conferring pod shattering resistance in another major podded crop, soybean. The impact of Sealicit^TM^ on soybean yield was assessed using a randomized block field trial design at two distinct geographical locations (Canada and Brazil) with four different varieties. Employing multiple experimental approaches, the impact of Sealicit^TM^ on plant and pod phenotypic traits and pod firmness were assessed for two Brazilian soybean varieties UFUS 6901 [shattering-susceptible (SS)] and UFUS 7415 [shattering-resistant (SR)] (Bicalho et al., 2019). In order to decipher the MOA at the molecular level, the expression of soybean pod shattering genes, *GmPDH1.1* and *GmSHAT1–5* were also evaluated. Overall, the current studies aimed to further support the efficacy of a specialty biostimulant from *A. nodosum*, with a defined composition, to reduce seed loss and increase yields in crops producing dry pods, bringing exciting opportunities for sustainable agriculture.

## Materials and Methods

### Study Site, Experimental Conditions, and Treatment Application

The field trials in Canada were conducted in 2017 on two commercial glyphosate herbicide-resistant varieties (McLeod R2 referred to as V1 and NSC Austin RR2Y referred to as V2 throughout the manuscript). V1 and V2 varieties were evaluated in Minto midwestern Ontario (located at 49°24′26″ N, 100°01′26″ W and 474-m altitude) and Elm Creek, Manitoba (located at 49°40′34″ N, 97°59′32″ W and 252-m altitude), respectively. V1 was seeded on May 17, 2017, and harvested on September 28, 2017. V2 variety was seeded on May 11, 2017, and harvested on September 29, 2017. The climate for both locations is classified as humid continental climate (Dfa) according the Köppen climate classification. A mean temperature of 15.8°C, ranging from 4.6 to 25.8°C, and a mean accumulated precipitation of 262.8 mm was recorded during the trial with V1 in Minto. The V2 trial was characterized by a mean temperature of 22.5°C, ranging from 5.2 to 27.5°C, and a mean accumulated precipitation of 284.5 mm. The soil type for clay loam and loamy sand for V1 and V2 field trials, respectively, and the previous crop in both sites was flax. V1 variety has an early cycle (124 days until harvest) with average *Sclerotinia* resistance and high seed yield potential (3.3 Mt/ha). V2 has an early cycle (124 days until harvest), superior white mold resistance, and high seed yield potential (3.2 Mt/ha). Soybeans were sown by mechanical drilling with a row space of 20 cm, at a seed rate of 140 and 100 kg/ha for V1 and V2, respectively. The pest management program consisted of the application of the herbicide Round-up (900 g/ha, Bayer) and the insecticide Matador (83 mL/ha; Syngenta).

Sealicit^TM^ is a proprietary PSI-759 ANE produced under high temperatures and alkaline conditions through a targeted PSI approach to formulation development and was provided by Brandon Bioscience (Tralee, Ireland). Sealicit^TM^ was applied at 1.5 L/ha by single foliar spray at vegetative stage V2–V3, which corresponds to the stage 14–16 according to the soybean BBCH scale (SoyBase, 2020). This system is used for uniform coding for phenologically similar growth stages of all monocotyledonous and dicotyledonous plant species (Hack et al., 1992). A portable CO_2_ spraying system at a constant pressure of 2.76 bars was used to apply an equivalent volume of 100 L/ha. Control plants were sprayed with an equal volume of water. The experimental design was a randomized block design with four replicates per condition and each plot was 9 m^2^.

The field experiments in Brazil were conducted at the FAFRAM experimental station located in the city of Ituverava-SP (20°00′00″ S, 47°47′20″ W and 631-m altitude) between December 2, 2019, and March 25, 2020. The climate is classified as a tropical wet and dry climate (*Aw*) according the Köppen climate classification. A mean temperature of 25.2°C, ranging from 13.6°C to 34.9°C, and a mean accumulated precipitation of 250.7 mm were recorded during the field trial period in the weather observing station INM759 of Ituverava [20°36′00″ S, 47°77′00″ W and 613-m altitude (Agritempo, 2020)]. The soil type was red clay latosol, and the previous crop grown in the field trial site was soybean. Both tested commercial soybean varieties were provided by the Soybean Improvement Program of the Federal University of Uberlândia (UFU, 2020). SS variety (UFUS 6901) has a very early cycle (108 days until harvest) with resistance to the nematode *Pratylenchus brachyurus* and high seed potential yield (3.9 Mt/ha). SR variety (UFUS 7415) has an early cycle (110 days until harvest) and is highly resistant to Asian soybean rust (*Phakopsora pachyrhizi*) with tolerance to drought stress and high seed potential yield (4.3 Mt/ha) (Hamawaki et al., 2018). Soybeans were sown by mechanical drilling with a row space of 50 cm, at a seed rate of 65.3 kg/ha, on December 2, 2019, with the fertilizer application of 340 kg/ha of formulated 04-14-08 (NPK). The pest management program consisted of the application of the broad-spectrum insecticide Kaiso (100 mL/ha; Nufarm), the post emergent herbicide Dribble (400 mL/ha; Sumitomo), and the systemic fungicide Fox (500 mL/ha; Bayer). Sealicit^TM^ was applied by single foliar spray at vegetative stage V5–V7, before the emergence of the inflorescence, which corresponds to stages 16–18 according to the soybean BBCH scale. Dosage rates were 0.75, 1.5, and 3.0 L/ha of Sealicit^TM^. A portable CO_2_ spraying system at a constant pressure of 2.1 bars was used to apply an equivalent volume of 200 L/ha. Control plants were sprayed with an equal volume of water. The distance between the spray lance and the plants was 50 cm, promoting a better cover of the sprayed biostimulant on the leaf tissue. The experimental design was a randomized block design in a 4 × 2 factorial design (number of treatments × number of varieties) with 13 replicates per condition (156 plots per field trial). The plots measured 2 m wide by 5 m long (10 m^2^) and had four rows. These rows were spaced 0.5 m; 0.5-m buffer zones were established for each plot, and only a central 4 m^2^ was used for phenotypic determinations or sampling purposes. The final plant stand per plot averaged 43 for both soybean varieties.

### Plant and Yield Measurements

In the Canadian trials, harvesting was performed using a calibrated plot combine, and final harvested plot yields were recorded for at least four independent biological replicates per variety and treatment with soybean seed yield extrapolated to kg/ha. In Brazil, plant and yield measurements were taken in at least eight independent biological replicates per variety and treatment. Three independent biological pod samples were collected per variety and treatment and pooled for further analysis. Plant height was measured from the surface of the soil to the end of the main stem (hypocotyl) at the stages of early flowering (R1; BBCH60) and full maturity (R8; BBCH89). Plant lodging was assessed visually according to the following scale (1–5); 1: almost all plants standing, 2: less than 25% plants show stem lodging, 3: 25–50% of plants show stem lodging, 4: 50–80% of plants show stem lodging, 5: 100% plants show stem lodging. The presence of Asian soybean rust disease pressure caused by the fungus *P. pachyrhizi* was assessed visually according a scale of one to five (1: no disease symptoms; 2: 25% plants with disease symptoms; 3: 50% plants with disease symptoms; 4: 75% plants with disease symptoms; 5: 100% plants with disease symptoms). Soybean plants at stage R8 were harvested manually and processed by a soybean threshing machine. The seed yield was determined from the central 4-m^2^ area of each plot. After weighing, the seed weight per plot was extrapolated to kg/ha.

### Evaluation of Pod Shattering Resistance and Pod Phenotypic Traits

Soybean plants from the trials in Brazil were used for determination of pod firmness and evaluation of phenotypic traits. Before harvesting (114 days after sowing), five plants at full maturity (R8 stage or BBCH89 when the fruit ripening is complete) were picked randomly from each plot. Pods were collected randomly from these plants. In contrast to *Arabidopsis* and oilseed rape, where a mechanical test is the most common technique to measure pod firmness (Morgan et al., 2001), the oven drying method is the most convenient and widely accepted assessment of pod shattering resistance in soybean. The test implemented during the course of these field trials was based on peer-reviewed published methods with minor modifications (Funatsuki et al., 2014; Krisnawati and Adie, 2017; Krisnawatiorcid et al., 2020). First, harvested pod samples were allowed to equilibrate their moisture levels at room temperature. After this acclimatization period, pod length was measured using ImageJ software (available at^1^) with a minimum of 60 biological replicates (pods) per variety and treatment. Evaluation of pod shattering resistance and seed weight per pod was performed on five biological replicates with 20 pods per replicate. Pod shattering resistance was analyzed after incubating pod samples at 65°C for the time required to open ≥ 50% of the control pods (Figure 2). This time period was calibrated for both SS and SR varieties. Pod shattering was calculated as the percentage of open pods versus total number of pods. After completing the pod shattering resistance test, the same dried soybean pods were used to determine the average seed weight per pod.

### RNA Isolation and qRT-PCR

RNA was obtained from soybean pods at stage R5 (BBCH 75–79), which corresponds to the stage 17b according to fruit developmental scale for the model plant *A. thaliana* (Smyth et al., 1990; Roeder and Yanofsky, 2006). Four green fruits per plant were collected from at least five plants per plot and immediately frozen in liquid nitrogen, ground, and stored in −80°C to prevent RNA degradation. All pods collected per plot were considered a single biological replicate. All samples were collected within 2 h of midday. For quantitative reverse transcriptase–polymerase chain reaction (qRT-PCR) analysis, at least three biological replicates of each treatment and variety were analyzed. Three technical replicates per biological replicate were analyzed. Expression analyses were performed by real-time PCR using a Roche LightCycler 96 System (Roche, United Kingdom). Quantitative PCR was performed using the LightCycler RNA Master SYBR Green I one-step kit (Roche, United Kingdom) according to the manufacturer’s instructions. The expression level of genes of interest was expressed in n-fold change and calculated according 2^–ΔΔCT^ (Livak and Schmittgen, 2001). Primers used for qRT-PCR reactions are as follows: *GmPDH1* FW: 5′-GAGGGAGGCGTTTTACGAC-3′; REV: 5′-GACGTGGCAACCATGACTC-3′ (Funatsuki et al., 2014); *GmSHAT1–5* FW: 5′-GGAGAACCACCACAACACCA-3′; REV: 5′-GTCCGTGCCCATCTCTACTG-3′ (AHJ81058.1); *GmCYP2* FW: 5′-CGGGACCAGTGTGCTTCTTCA-3′; REV: 5′-CCCCTCCACTACAAAGGCTCG-3′ (Jian et al., 2008). *GmCYP2* was used as the reference gene for normalization.

### Statistical Analysis

Statistics were evaluated with Sigma Plot 12 and Statgraphics Centurion XVI software. The seed yield differences between control and Sealicit^TM^ treatment for V1 and V2 were analyzed using *t* test at *p* ≤ 0.05. The effects of Sealicit^TM^ on pod shattering, *GmPDH1* and *GmSHAT1–5* gene expression was analyzed with one-way analysis of variance (ANOVA) by Tukey honestly significant difference (HSD) test at *p* ≤ 0.05. The rest of plant data were compared by using two-way ANOVA, with Tukey’s HSD test at *p* ≤ 0.05. Where the interaction between the two factors, variety (SS and SR) and Sealicit^TM^ treatment (0, 0.75, 1.5, and 3 L/ha) (V × S), was significant, data were subjected to one-way ANOVA, comparing all Sealicit^TM^ treatments with each other within the same soybean variety. Where V × S interaction was not significant, the effects of variety and Sealicit^TM^ treatments were evaluated separately, comparing the respective means through *t* test (variety) or one-way ANOVA Tukey’s HSD test (Sealicit^TM^ treatment) at *p* ≤ 0.05. The application of all parametric tests was performed after checking the normality of the data (Shapiro–Wilk test) and equal variance assumptions. Unless stated otherwise, all data are expressed as average ± standard error (SE). Details of the individual sample size for each analysis are mentioned in the tables and figure legends.

## Results

### Effects of Sealicit^TM^ on Soybean Seed Yield

**FIGURE 1:**
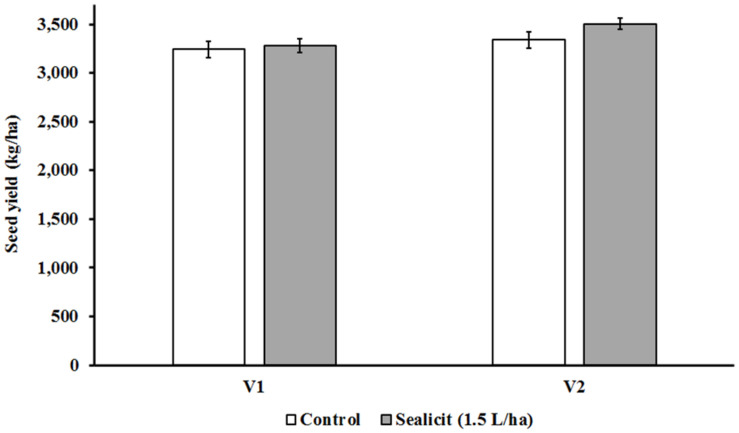
Effects of Sealicit^TM^ on seed yield in two commercial soybean varieties with glyphosate herbicide resistance. Sealicit^TM^ was applied by foliar spray (1.5 L/ha) at vegetative stage (V2–V3), and its performance was benchmarked against untreated plants. Field trials with V1 and V2 were carried out in Minto (ON, Canada) and Elm Creek (MB, Canada) in 2017 under standard grower practice, respectively. Each bar represents seed yield (kg/ha). Number of biological replicates (*n* = 4).

The Canadian trial seed yields for control plots of both tested varieties (V1: 3,241 kg/ha; V2: 3,388 kg/ha) were similar to average values obtained by Canadian growers in the same growing season (V1: 3,256 kg/ha; V2: 3,188 kg/ha) (Manitoba Pulse Soybean Growers, 2017). The application of Sealicit^TM^ in V1 at vegetative stage did produce a small statistically nonsignificant yield increase (+1.08%; *p* = 0.796). However, the V2 variety had a larger increase in yield of 4.91% (*p* = 0.207), producing 164 kg/ha more than control plots (Figure 1). The impact of Sealicit^TM^ on yield in the Brazilian trials was also evaluated. A two-way ANOVA analysis demonstrated that Sealicit^TM^ treatment had a statistically significant effect on seed yield (*p* = 0.026). This parameter was also highly affected by the interaction variety x Sealicit^TM^ (*p* = 0.047) (Table 1). The SS variety had increased seed yield of 6.6% (*p* = 0.078) and 6.2% (*p* = 0.154) at low (0.75 L/ha) and high (3 L/ha) doses, respectively. Only a minor increase of 2.2% (*p* = 0.555) was recorded at the 1.5 L/ha rate for the SS variety. Sealicit^TM^ provided a more pronounced positive yield effect on the SR variety when it was applied at low (13.1%; *p* = 0.047) and medium (17.9%, *p* = 0.016) doses, respectively. However, only a small increase was recorded for the high Sealicit^TM^ dose (1.9%; *p* = 0.774).

**TABLE 1:** Analysis of variance and mean comparisons for fresh seed weight per pod, pod length, and harvested seed yield at full maturity stage (R8) with two commercial soybean varieties treated with water and three concentrations of Sealicit^TM^.

Source of variance	Seed weight/pod (g)	Pod length (cm)	Seed yield (kg/ha)
Variety (V)	NS	***	NS
Sealicit^TM^ (S)	*	***	*
V × S	NS	NS	*
**Variety**			
SS	0.44 ± 0.01	4.04 ± 0.02a	3,691.4 ± 63.6
SR	0.44 ± 0.01	4.14 ± 0.02b	3,773.3 ± 66.3
**Sealicit^*TM*^**			
Control	0.42 ± 0.01a	4.03 ± 0.02a	3,522.2 ± 93.8*a*
0.75 L/ha	0.45 ± 0.01b	4.13 ± 0.02b	3,867.1 ± 86.9*b*
1.5 L/ha	0.43 ± 0.01*ab*	4.03 ± 0.02a	3,873.7 ± 86.9*b*
3 L/ha	0.46 ± 0.01b	4.18 ± 0.02b	3,666.4 ± 99.5*a*
**V × S**			
SS + control	0.40 ± 0.02	3.98 ± 0.03	3,558.4 ± 63.7a
SS + 0.75 L/ha	0.46 ± 0.01	4.13 ± 0.04	3,792.0 ± 79.4ab
SS + 1.5 L/ha	0.43 ± 0.01	3.94 ± 0.03	3,636.3 ± 59.8a
SS + 3 L/ha	0.47 ± 0.02	4.13 ± 0.03	3,779.0 ± 147.0a
SR + control	0.43 ± 0.01	4.09 ± 0.03	3,486.0 ± 169.9a
SR + 0.75 L/ha	0.44 ± 0.01	4.12 ± 0.04	3,942.2 ± 170.6b
SR + 1.5 L/ha	0.43 ± 0.01	4.11 ± 0.03	4,111.2 ± 111.4b
SR + 3 L/ha	0.45 ± 0.00	4.23 ± 0.03	3,553.8 ± 125.0*a*

NS, *, and ***, nonsignificant or significant at p ≤ 0.05, and p ≤ 0.001, respectively. Different letters indicate statistical differences for p ≤ 0.05 based on t test (V) or one-way ANOVA Tukey’s HSD test (S or V × S). Number of biological replicates (seed weight, n = 5; pod length, n ≥ 60; seed yield, n ≥ 8). ±represents standard error (SE).

### The Effects of Sealicit^TM^ Treatment on Soybean Plant Development

The impact of soybean variety and Sealicit^TM^ treatment on a number of plant phenotypic parameters including height, degree of lodging, and the prevalence of Asian soybean rust was assessed in both Brazilian varieties. A two-way ANOVA test revealed that in conjunction both parameters (variety × Sealicit^TM^) had no significant effect on height or lodging parameters (Table 2). The SS plants showed an overall statistically significant increase of plant height at flowering (+9.2%; *p* = 0.006) and full maturity (+9.8%; *p* ≤ 0.001) stage compared to SR plants. SS plants had increased their lodging degree 1.6 times compared to SR plants (*p* ≤ 0.001); however, the absolute values of this parameter confirmed that less than 25% of the soybean plants were not erect. The effect of Sealicit^TM^ on plant height and the degree of lodging were not statistically significant with respect to the control. Finally, soybean plants did not show any symptoms of Asian rust disease indicating the quality of the field trial (Table 2).

**TABLE 2:** Analysis of variance and mean comparisons for plant height at flowering (R1) and full maturity (R8) stage, lodging degree, and degree of Asian rust disease with two commercial soybean varieties, treated with water and three concentrations of Sealicit^TM^.

Source of variance	Plant height flowering (cm)	Plant height maturity (cm)	Lodging degree (1–5)	Asian rust disease degree (1–5)
Variety (V)	**	***	***	NS
Sealicit^TM^ (S)	NS	NS	NS	NS
V × S	NS	NS	NS	NS
**Variety**				
SS	84.2 ± 1.8^b^	95.1 ± 1.8^b^	2.2 ± 0.2^b^	1.0 ± 0.0
SR	77.1 ± 1.8^a^	86.6 ± 1.5^a^	1.4 ± 0.1^a^	1.0 ± 0.0
**Sealicit^TM^**				
Control	81.4 ± 2.3	91.4 ± 2.5	1.7 ± 0.2	1.0 ± 0.0
0.75 L/ha	80.8 ± 2.8	91.2 ± 2.8	1.8 ± 0.3	1.0 ± 0.0
1.5 L/ha	81.6 ± 2.2	91.5 ± 2.3	1.8 ± 0.3	1.0 ± 0.0
3 L/ha	78.8 ± 2.7	89.3 ± 2.8	1.8 ± 0.2	1.0 ± 0.0
**V × S**				
SS + control	82.0 ± 2.8	93.3 ± 2.8	2.0 ± 0.4	1.0 ± 0.0
SS + 0.75 L/ha	82.6 ± 4.6	92.6 ± 4.5	2.1 ± 0.4	1.0 ± 0.0
SS + 1.5 L/ha	87.6 ± 2.5	98.4 ± 2.5	2.4 ± 0.4	1.0 ± 0.0
SS + 3 L/ha	82.4 ± 3.7	93.7 ± 3.7	2.0 ± 0.4	1.0 ± 0.0
SR + control	80.8 ± 3.7	89.6 ± 3.9	1.4 ± 0.2	1.0 ± 0.0
SR + 0.75 L/ha	76.9 ± 2.3	87.4 ± 2.2	1.3 ± 0.2	1.0 ± 0.0
SR + 1.5 L/ha	75.7 ± 2.4	84.6 ± 1.8	1.2 ± 0.1	1.0 ± 0.0
SR + 3 L/ha	75.1 ± 3.6	85.0 ± 3.6	1.7 ± 0.2	1.0 ± 0.0

NS, **, and ***, nonsignificant or significant at p ≤ 0.01, and pp ≤ 0.001, respectively. Different letters indicate statistical differences for p ≤ 0.05 based on t test (V) or one-way ANOVA Tukey’s HSD test (S or V × S). Number of biological replicates (n ≥ 8). ± represents standard error (SE).

### Effects of Sealicit^TM^ on Pod Morphology

In order to assess whether Sealicit^TM^ had any impacts on soybean pod growth and development, as reported previously for *Arabidopsis* and WOSR (Łangowski et al., 2019), the average fresh seed weight per pod and pod length were measured in SS and SR varieties. A two-way ANOVA revealed that Sealicit^TM^ had a significant effect on the average fresh seed weight per pod (*p* = 0.024) (Table 1). In comparison to the control, the average fresh seed weight was the highest at 0.75 L/ha with the 3 L/ha application rate (7.1 and 9.5%), followed by a lower yet significant increase at the 1.5-L/ha application rate (2.4%). The two-way ANOVA test did not show any significant interactions across varieties and Sealicit^TM^ treatments on pod length (*p* = 0.060). However, there was a significant increase in pod length in the SR, when compared to the SS variety (*p* ≤ 0.001). When these differences were examined in detail, there were also significant differences between the treated and untreated plants (*p* ≤ 0.001). Similarly, to seed weight per pod, the low and higher application rates, for both tested varieties, positively affected the pod length, which was longer by 2.5 and 3.4% as compared to control, respectively (Table 1).

### Effects of Sealicit^TM^ on Pod Shattering Resistance

**FIGURE 2:**
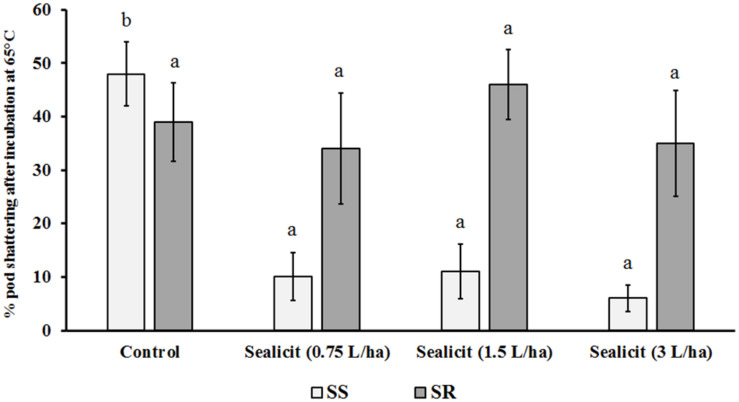
Effects of Sealicit^TM^ rate on soybean pod shattering. Each bar represents the percentage of open pods versus total number of pods. Different letters within the same soybean variety indicate statistically significant differences between the treatments based on one-way ANOVA Tukey’s HSD test at *p* ≤ 0.05. Number of biological replicates (*n* = 5).

The effect of Sealicit^TM^ and control treatments on the shatter resistance of harvested pods from the Brazilian field trial was assessed. As expected, the SS variety required a shorter calibration time (4 h) compared to the SR variety (48 h), demonstrating the sensitivity of the method used. Interestingly, when applying the same incubation times for the treated samples, we observed a strong effect of Sealicit^TM^ on SS with very few pods bursting compared to the controls across all treatments (*p* ≤ 0.001). This suggested that the lowest dose (0.75 L/ha) was sufficient to confer shattering resistance to the susceptible variety by decreasing pod opening by nearly fivefold. The SR samples showed minor decreases in shatter at low and high doses, but a slight increase at 1.5 L/ha dose; however, these fluctuations were not statistically significant (Figure 2).

### Sealicit^TM^ Affects the Development of Dehiscence Zone

**FIGURE 3:**
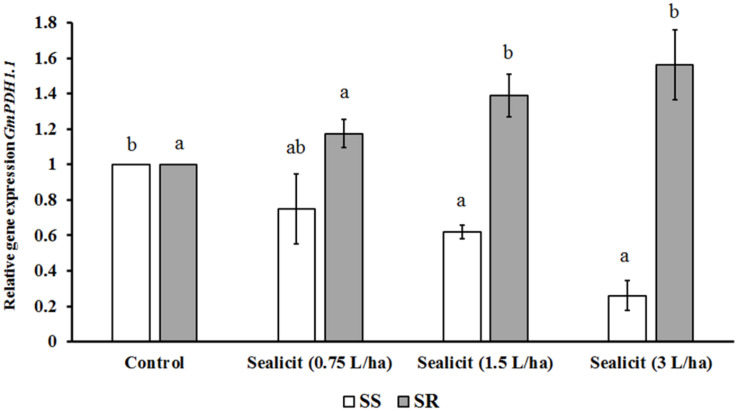
Relative expression of *GmPDH1.1* in soybean pods treated with Sealicit^TM^. Different letters within the same soybean variety indicate statistically significant differences between the treatments based on one-way ANOVA Tukey’s HSD test at *p* ≤ 0.05. Number of biological replicates (*n* = 3).

**FIGURE 4:**
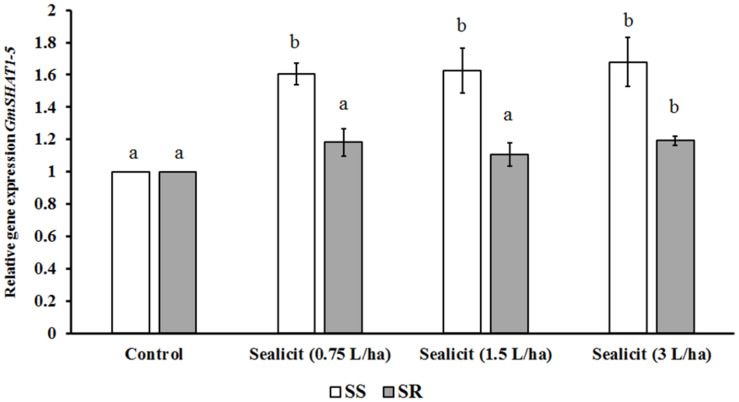
Relative expression of *GmSHAT1–5* in soybean pods treated with Sealicit^TM^. Different letters within the same soybean variety indicate statistically significant differences between the treatments based on one-way ANOVA Tukey’s HSD test at *p* ≤ 0.05. Number of biological replicates (*n* = 3).

To further assess the impact of Sealicit^TM^ on pod shatter, the expression level of two major genes regulating pod dehiscence, *GmPDH1.1* (active in inner sclerenchyma) and *GmSHAT1–5* (active in FCCs), was examined (Funatsuki et al., 2014). The relative gene expression change was measured in reference to the housekeeping gene *CYCLOPHILIN2* (*CYP2*) (Jian et al., 2008). Funatsuki et al. (2014) showed that the transcript level of *GmPDH1.1*, which is involved in secondary wall biosynthesis and lignin deposition, decreased due to an early stop codon mutation, which is characteristic for pod-shatter resistant varieties. In our current studies, we observed that Sealicit^TM^ significantly and gradually decreased *GmPDH1.1* expression in a concentration-dependent manner, in the variety SS (Figure 3). However, the expression analysis performed in the SR variety showed the opposite trend, which supports a variety-dependent effect. Next, the expression level of *GmSHAT1–5*, which similarly to *GmPDH1* is involved in secondary wall biosynthesis, was examined. Interestingly, the transcript level in pods collected from treated plants was increased in both varieties (Figure 4). The SR variety had a small increase that was statistically significant at the highest dose. On the other hand, a significant increase (1.6-fold) was observed for the SS variety, which is in agreement with *GmPDH1* expression level and the pod firmness test (Figure 3).

## Discussion

Soybean (*G. max*) is one of the most important global crops because of its high protein and fat content. Its broad use for the production of food, oil, and fodder creates a continuously growing demand, having serious implications for the natural environment (Kocira et al., 2019). In order to reduce the impact of agricultural-related deforestation and pollution, new solutions for more efficient and sustainable soybean production are urgently needed. In our previous work, we have shown that ANE biostimulant Sealicit^TM^ is capable of reducing pod shattering, influencing fruit development and yield in the model plant system *Arabidopsis*, as well as selected varieties of WOSR (Łangowski et al., 2019). Following on from the previous detailed analysis of Sealicit^TM^ action, we have tested whether similar effects can be observed in soybean grown in field conditions. In this study, the impact of Sealicit^TM^ on soybean productivity and pod shattering, as assessed by (i) testing pod-shatter in low humidity conditions and (ii) analyzing the expression level of major genes determining pod shattering, was determined. Productivity parameters were complemented with morphological analysis of plant height, seed weight per pod, pod length, and finally seed yield assessment. Here, we demonstrated that the same biostimulant can be successfully utilized not only in the Brassicaceae family species but also legumes, providing a new solution for a pressing problem.

### Soybean Yield Evaluation

Sealicit^TM^ field testing in Canada and Brazil using a randomized block field trial design showed consistent and agronomically sound yield results (Table 1 and Figure 1). V1 and V2 seed yield values in control plots were very similar to average values obtained by Canadian growers in the same growing season (V1: 3,256 kg/ha; V2: 3,188 kg/ha, respectively) (Manitoba Pulse Soybean Growers, 2017). The effect of Sealicit^TM^ appears to be variety specific, which is similar to that previously observed for WOSR. The yield increase for V2 was 4.91% with a *p* = 0.207. Although the *p* value did not meet the typical 0.05 scientific threshold, the magnitude of the yield increase was agronomically interesting, and it provided a basis for running additional trials with a larger number of replicates to reduce the *p* value. It should be noted that little information is available on the susceptibility of these short-cycle Canadian varieties to pod shatter, so additional trials were performed with soybean varieties with known shatter susceptibility to investigate the potential of Sealicit^TM^ in this crop. The harvested seed yields at full maturity for the Brazilian varieties SS and SR were in the range expected in previous field trials developed in four consecutive sowing seasons in Uberlândia-MG (Bicalho et al., 2019). All treated soybean varieties with Sealicit^TM^ produced increased yield with respect to their untreated counterparts. Yield increases were statistically significant with *p* ≤ 0.05 for treatments, likely due to the higher replicate numbers versus the Canadian trials. It is evident that there was a significant interaction between the dose (0.75 and 1.5 L/ha) and the yield for the SR variety, which was not the case for the SS variety. Therefore, dose optimization on specific varieties may be important to gain maximum benefit from Sealicit^TM^ in soybean crops.

### Impact of Sealicit^TM^ Treatments on Soybean Architecture and Pod Phenotype

While the SS variety was significantly taller than the SR variety, Sealicit^TM^ did not demonstrate an ability to influence plant height. From published work on ANEs in soybean and tomato plants, we have learned that this class of plant biostimulants did not have a statistically significant effect on plant height despite yield increase (Guerreiro et al., 2017; Carmody et al., 2020). It indicates that this phenotypic trait may not be sufficient to predict yield benefits provided by ANE biostimulants. We have previously reported that Sealicit^TM^ is able to increase the pod weight in *Arabidopsis* and seed weight of some WOSR varieties (Łangowski et al., 2019). Here we observed a substantial seed weight increase in both soybean varieties tested. This result is contrary to data published by Kocira et al. (2019), which presents a 1,000-soybean-seed weight decrease after foliar spray with a nonspecialty seaweed extract from *Ecklonia maxima*. This finding highlights the importance of the seaweed specie and the process used for extraction on bioactivity-related parameters of seaweed extract biostimulants (Goñi et al., 2016, 2018; Shukla et al., 2019; Carmody et al., 2020). Moreover, both soybean varieties showed an increased pod length, which is in agreement with Sealicit^TM^ ability to dysregulate *FUL*, *RPL*, and *IND* expression and pod length in *Arabidopsis* and some WOSR varieties (Łangowski et al., 2019), suggesting that this specialized ANE biostimulant may also modulate soybean pod auxin homeostasis and signaling, crucial for fruit growth and development (Liljegren et al., 2004; Sorefan et al., 2009; Dong et al., 2019).

### Sealicit^TM^ Impact on Pod Shattering Resistance and Pod Dehiscence Genes

Data derived from multiple trials in commercial WOSR varieties demonstrated a positive association between pod firmness and yield (Łangowski et al., 2019). Soybean pod architecture and dehiscence mechanism show significant differences in relation to Brassicaceae fruits, yet the principle of seed dispersal is still relying on lignin deposition that determines physical resistance (Dong et al., 2014; Funatsuki et al., 2014). Pod shattering resistance tests performed on soybean control pods showed variability in treated varieties, ranging from 4 h for SS to 48 h for SR, proving the robustness and sensitivity of the method. The difference in calibration time was to be expected as both varieties are genetically diverse (Bicalho et al., 2019). On the other hand, the SR variety, classified as resistant to shatter, showed no statistically significant improvement after Sealicit^TM^ treatments, whereas the SS variety, classified as susceptible to pod shattering, showed a very significant fruit firmness increase across all the doses applied. In addition, Sealicit^TM^ decreased *GmPDH1.1* and increased *GmSHAT1–5* expression in the SS variety, which is consistent with a reduction in shatter. Moreover, *GmPDH1.1* transcript levels were decreasing with increasing Sealicit^TM^ concentration in SS variety, yet increasing in SR, indicating that the effect is dose and genome/variety dependent. It is important to note that the SR variety, despite showing insignificant increase in *GmPDH1.1* transcript level, also showed slight increase in *GmSHAT1–5*. Taking into account that the transcript levels of both might be extremely different, a small increase in SR *GmSHAT1–5* transcript could not only counteract the insignificant increase of *GmPDH1*, but also account for a small increase of SR in pod firmness test (Figure 2). Therefore, it is tempting to speculate that pod firmness in soybean fruit is a balance between the amount of the lignin deposited in the FCCs and inner sclerenchyma, which may be determined by multiple genes that ultimately regulate the dorsoventral tension. In order to gain an in-depth understanding of the mechanism and critical signaling pathways, transcriptome and proteome analysis of multiple genes involved in fruit development (i.e., *FUL*, *RPL*, *IND*, *SHP*, and *ALC*) (Ferrándiz, 2002), their mutants, and complementation lines are necessary (Dong and Wang, 2015). Most importantly, the enhanced productivity of SS was positively associated with *GmPDH1.1* and *GmSHAT1–5* gene expression, increased pod shattering resistance, and higher seed weight per pod. However, the seed yield increase in treated SR soybean plants was not clearly associated with any of the parameters evaluated in this study, being likely due to additional effects promoted by Sealicit^TM^ that have not been the subject of investigation. As observed previously by Guerreiro et al. (2017), foliar applications of ANE biostimulant can stimulate additional yield-related parameters in soybean such as increased number of pods per plant, which could be one of the contributing factors. Briglia et al. (2019) also showed an improvement in soybean biovolume and green area after foliar spray treatment with different combinations of seaweed and plant extracts. Those additional benefits of ANE application could potentially account for increased seed weight and fresh seed yield in the SR variety.

### Agronomical and Environmental Implications

The gradually increasing soybean production (by 4–5% annually) in Brazil means it will become the biggest world producer with an estimated 129 Mt in season 2020/2021. The harvested area is forecasted at a record 36.8 Mha, up by 1.7 Mha from last season’s record (USDA, 2020). While soybean production in Brazil is growing, losses due to adverse weather conditions become more frequent (Rowntree et al., 2013; Van Roekel et al., 2015; Zhang et al., 2018). The Brazilian Agricultural Research Corporation (EMBRAPA) has established that one 60-kilo bag per hectare is an acceptable loss threshold value (Silveira et al., 2019). However, in the last 30 years, a wide range of yield losses (54–561 kg/ha) has been recorded in different regions of Brazil due to adverse weather events and delayed and/or inadequate mechanical harvesting (Chioderoli et al., 2012; Barbosa and Schmitz, 2015; Bandeira, 2017), averaging 120 kg/ha loss at a national level (Schanoski et al., 2011). In this study, we have demonstrated that this lost yield can be recovered and safeguarded using the ANE biostimulant Sealicit^TM^ at a low dose rate (0.75 L/ha), increasing seed yield by an average of 9.8% in two commercial soybean varieties (+344.9 kg/ha). The use of the biostimulant would allow production output to grow in a sustainable manner contrary to expanding crop area. It is important to note that the yield uplift that can be obtained with Sealicit^TM^ application provides a compelling return on investment for the grower (>3:1). According to the current average market price of soybean, growers will be able to obtain added revenues of $135/ha by applying Sealicit^TM^ in the Brazilian market, which creates a strong financial incentive for the growers to switch to a more environment-friendly and sustainable agriculture.

### Conclusions and Future Perspectives

ANE-based biostimulants are a rapidly growing crop input, because of their efficacy in a number of applications in modern agriculture. The investigations reported were undertaken to determine if Sealicit^TM^ has an impact on pod development in soybean. The data generated suggest that Sealicit^TM^ has a strong antishattering effect in a non–shatter-resistant soybean variety. It is also increasingly evident that Sealicit^TM^ treatments, despite impacting on pod dehiscence, may have in some species and varieties accompanying effects stimulating average seed weight per pod and pod length, which ultimately reflects on yield. In order to gain a better understanding of Sealicit^TM^’s MOA in soybean, lignin staining and transversal sections presenting fruit morphology and lignin deposition could be further analyzed. Pod shattering is determined by multiple contributing genes, which makes their identification challenging even by GWAS. Therefore, a lack of sufficient understanding of physiological and genetic mechanisms hinders improvements of such traits by intensive breeding or genome editing (Verdugo et al., 2010; Korte and Farlow, 2013). Sealicit^TM^ appears to provide a solution for a yield reducing concealed developmental trait that is pod shattering, not only in oilseed rape but also in soybean and possibly other species relying on similar, conserved, seed dispersal mechanisms. Biostimulants that mobilize genetic plant potential to achieve high-quality crop with maximum yield represent an attractive tool to currently applied methods for efficient food production.

## Data Availability Statement

The original contributions presented in the study are included in the article.

## Author Contributions

ŁŁ, OG, JG, OH, and SO’C conceived and designed the experiments. ŁŁ, FM, MT, MP, CS, and AN performed the experiments. ŁŁ, OG, OH, FM, and SO’C analyzed the data. ŁŁ, OG, and SO’C wrote the article. All authors contributed to the article and approved the submitted version.

## Conflict of Interest

The authors declare that the research was conducted in the absence of any commercial or financial relationships that could be construed as a potential conflict of interest.

## Footnotes

1 https://imagej.net/Welcome
